# The effect of mid-season coach turnover on running match performance and match outcome in professional soccer players

**DOI:** 10.1038/s41598-022-14996-z

**Published:** 2022-06-23

**Authors:** Łukasz Radzimiński, Alexis Padrón-Cabo, Toni Modric, Marcin Andrzejewski, Sime Versic, Paweł Chmura, Damir Sekulic, Marek Konefał

**Affiliations:** 1 Department of Physiology, Gdansk University of Physical Education and Sport, K. Górskiego 1, 80-336 Gdańsk, Poland; 2 Department of Physical Education and Sport Science, University of A Coruña, 15071 A Coruña, Spain; 3 Faculty of Kinesiology, University of Split, 21000 Split, Croatia; 4 Department of Recreation, University School of Physical Education, Poznań, Poland; 5 Department of Team Games, University School of Physical Education, Wrocław, Poland; 6 Department of Biological and Motor Sport Bases, University School of Physical Education, Wrocław, Poland

**Keywords:** Psychology and behaviour, Human behaviour

## Abstract

The aim of this research was to examine the physical match performance and match outcome before and after coach turnover in professional soccer teams compared with a control group containing coaches working continuously for 3 consecutive seasons. Analysed data included 325 performances of teams led by dismissed coaches (DC), 313 of teams led by new coaches (NC), and 580 of teams led by unchanged coaches (UnC). Variables, such as average number of collected points, total distance (TD), total distance per minute (TD/min), high-speed running (HSR), sprinting and number of high-intensity runs (NHIR), were analysed in the last 15 games of DC and 15 matches of NC. These periods were divided into 3 blocks out of 5 matches (5-match blocks). NC collected a significantly higher number of points than DC (*p* = 0.015), whereas average points collected by UNC were significantly greater than DC (*p* < 0.001 and NC (*p* = 0.012). TD and TD/min for DC were significantly lower (*p* < 0.001) compared with both NC and UnC, whereas significant differences in HSR (*p* = 0.003) and NHIR (*p* = 0.03) were identified between DC and NC. The current study reported that mid-season coach turnover may result in short-term improvement in team results and physical match performance. However, this effect disappears after a period of approximately 5 games.

## Introduction

Worldwide observations indicate that the coach turnover rate in professional soccer is very high^1,2^. Despite the fact that sacking the coach from elite clubs causes considerable disturbances in player and team development, organizational growth and financial safety^3^, club management still decides to replace coaches during the season. Van Ours and Van Tuijl^4^ claimed that these changes are often caused by the dissatisfaction of stakeholders, sponsors and fans; the impact of media or—insufficient results. However, the most common reasons for coach turnover are recent match results and unsatisfactory position in the league^5,6^. This finding is not surprising since success in professional soccer is highly dependent on the obtained results^7^. The systematic victory of the subsequent games allowed the team to occupy a higher position in the league table and hence to win the trophies. On the other hand, getting trophies usually improves the clubs’ financial situation as well^8^. Therefore, coaches should take into account that after a certain number of lost matches, club authorities may decide to change them^4^.

Regardless of the type of sport and age category, the head coach is a crucial person responsible for team results and players’ development^9,10^. Previous studies highlighted that soccer coaches may influence the performance of their players^11^. Some authors demonstrated that coach encouragement may be a useful tool for manipulating the internal and external training load^12,13^. Ekstrand et al.^14^ reported that the leadership style of soccer coaches is associated with the incidence of severe injuries. All these findings emphasize the importance of the head coach role in planning and implementing the technical, tactical and physical aspects of the training process.

The final match results in soccer may be influenced by numerous variables, such as technical, tactical and physical performance^15–17^. The association between match outcome and physical performance at different intensities was previously established in elite soccer players^18^. Andrzejewski et al.^19^ emphasized the importance of match outcome when assessing the physical aspects of soccer performance. Concretely, Modrić et al.^20^ demonstrated that depending on the playing position, locomotion variables, such as running distance (14.4–19.7 km/h), sprint distance (> 25.2 km/h), decelerations (< − 0.5 m/s^2^) and high-intensity accelerations (> 3 m/s^2^), may significantly affect real game outcome. Therefore, physical activity during the match may indirectly contribute to the results obtained by each team and consequently to the decision to change the coach.

In the available literature, several studies have investigated the influence of coach turnover during the competitive season in soccer. Approximately one decade ago, such longitudinal analyses were performed for soccer leagues in Spain^21^ and Germany^22^. Lago-Penas^21^ showed that the short-term positive impact of coach turnover on team performance is followed by continued gradual worsening of further match results. Similarly, Heuer et al.^22^ found that dismissing the coach in the mid-season has no effect on the subsequent results of the team. Most recently, Zart and Gullich^23^ demonstrated that an in-season head coach change may result in instant performance improvement that could remain for up to 16 matches. Although these studies have provided valuable insights into match performance after coach turnover during the competitive season in soccer, knowledge of how coach turnover impacts physical performance remains limited.

The impact of coach turnover on physical match performance has not been thoroughly examined. To date, only the study of Guerrero-Calderon et al.^24^ has demonstrated how coach turnover affects physical responses both in training and competition. Interestingly, some reductions in both training and match physical performance were observed in this research. However, in this paper, the authors analysed only short-term effects in individual teams from the 3 highest leagues of Spain. Therefore, evaluating the short- and long-term impact of new coaches on match locomotion seems to be an important topic. Such analyses could provide numerous information for club authorities about potential effects of such personal changes. The main purpose of this research was to compare physical match performance and match outcome before and after coach turnover in professional soccer teams. To the best of our knowledge, this is the first study comparing match outcome and physical match performance between dismissed coaches and new coaches with reference to a control group (coaches that were not replaced during 3 consecutive seasons).

## Methods

### Study design

During three consecutive seasons (2018/2019, 2019/2020 and 2020/2021) of Polish Ekstraklasa (highest level of competition), 27 mid-season coach turnovers occurred. However, in 4 cases, coaches led their teams in fewer than 10 matches. Therefore, the performances of these teams were excluded from the data analysis. Moreover, 6 changes in coaches that were implemented between the seasons were not taken into consideration. The information about the coach turnovers were collected from official clubs announcements. The team performance data for each analysed club were split into a period when the team was led by a coach who was going to be dismissed (dismissed coaches—DC) and into a period when the team was led by a new coach (NC). Based on previous research^21,23^, for each DC, a group of the last 15 matches was analysed (these games were defined as matches between − 15 to − 1). Identically, the first 15 games of NC were taken into consideration (these matches were numbered from 1 to 15). Additionally, a total of 15 games were divided into 3 blocks out of 5 matches (for DC: period I contained matches from − 15 to − 11; period II from − 10 to − 6; period III from − 5 to − 1; for NC: period IV: first 5 matches; period V contained matches from 6 to 10; period VI from 11 to 15). During the analysed period of time, 6 coaches of Ekstraklasa clubs continued their work without any interruption (unchanged coaches—UnC). The results obtained by 6 teams led by UnC were considered the control group. All the data were anonymized in accordance with the Declaration of Helsinki. This study was approved by the Senate Committee on Ethics of Scientific Research at the Academy of Physical Education in Wroclaw (reference number: 12/2021). The Senate Committee also waived the need of collecting the informed consent forms.

### Data collection

A total of 1118 Polish Ekstraklasa (highest level of competition in Poland) team performances from 2018/2019 to 2020/2021 were analysed in the study. This total included the data of 325 team performances led by DC, 313 team performances led by NC, and 580 team performances led by UnC. The data regarding the number of collected points (won game—3 points, draw—1 point, lost game—0 points) and running performance during the matches were analysed. The locomotion variables during the matches were registered using a previously validated computerised multiple-camera optical tracking system TRACAB® (ChryronHego VID, New York, NY) with a sampling frequency of 25 Hz^25^. The match running performance contained the following variables: total distance covered (TD), total distance per minute (TD/min), high-speed running distance (HSR, 19.8–25.1 km∙h^−1^), and sprinting distance (> 25.2 km∙h^−1^). The presented division of the speed zones was consistent with that reported by Di Salvo et al.^26^. The total number of high-speed runs and total number of sprints were defined as the number of high-intensity runs (NHIR).

### Statistical analysis

The data are presented as the means ± standard deviations (SD) with 95% confidence intervals (95% CI). The datasets were analysed using the Shapiro–Wilk test for normal distributions. One-way Analysis of variance (ANOVA) was performed to identify potential differences between teams’ locomotion. Pairwise comparisons were conducted via Tukey’s post-hoc test for unequal samples. For the variables where data were not compatible with a normal distribution (number of collected points and sprint distance), possible differences were investigated using the nonparametric Kruskal–Wallis H test and the Mann–Whitney U test. The effect size (ES) for significant differences was determined using Cohen’s *D*^27^. According to Hopkins et al.^28^, the ES was classified as trivial (< 0.2), small (> 0.2–0.6), moderate (> 0.6–1.2), large (> 1.2–2.0) and very large (> 2.0–4.0). All analyses were performed using STATISTICA software version 13.0 (TIBCO Software, Inc., 2017). The significance level was set at *p* < 0.05.

## Results

**Figure 1:**
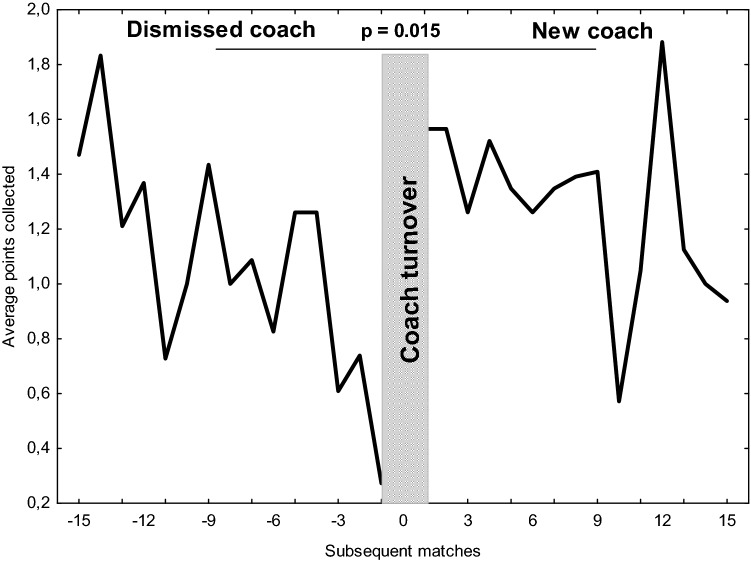
Average points collected by dismissed coaches during their last 15 matches and new coaches in their first 15 games.

**Figure 2:**
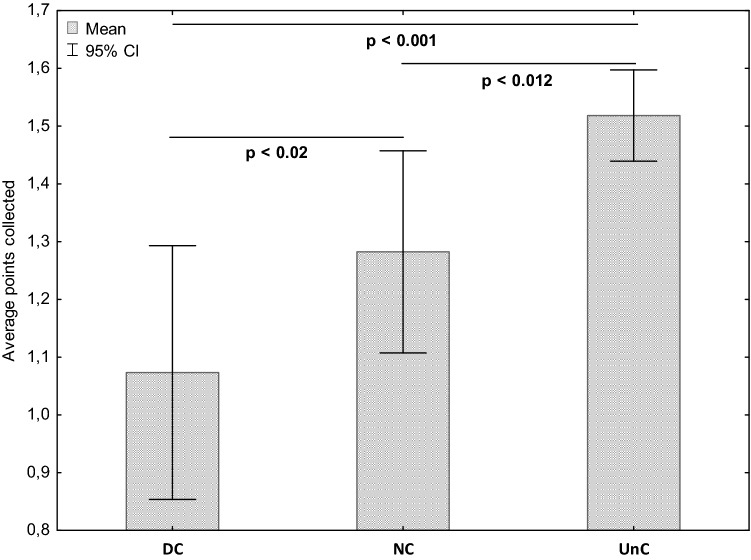
Comparison of average points collected by dismissed coaches (DC), new coaches (NC), and unchanged coaches (UnC).

Figure 1 illustrates the mean achieved points for every round before and after the coach turnover. The average number of points collected by DC (1.05 ± 1.26 points) in their last 15 games was significantly lower than that obtained by NC (1.29 ± 1.31 points, *p* = 0.015, ES = 0.20) and UnC (1.52 ± 1.32 points, *p* < 0.001, ES = 0.37). Moreover, UnC collected significantly more points than NC (*p* = 0.012, ES = 0.18) (Fig. 2).

The statistical analysis of physical match performance revealed that both NC and UnC covered significantly longer total distance (*p* < 0.001, ES = 0.30 and 0.43, respectively) and relative total distance (*p* < 0.002, ES = 0.28 and 0.40, respectively) compared with teams led by DC. Furthermore, greater HSR distance (*p* < 0.003, ES = 0.27) and NHIR (*p* < 0.03, ES = 0.21) were noted after coach turnover. No significant differences in sprinting were observed among DC, NC and UnC. The physical match activity of teams led by NC and UnC did not differ significantly (Table 1).

**Table 1:** Comparison of the physical match performance of the teams led by coaches who are going to be dismissed (DC), new coaches (NC) and unchanged coaches (UnC).

	DC	NC	UnC
TD (km)	111.7 ± 4.37	113.0 ± 4.26*	113.6 ± 4.39*
TD/min (m/min)	1155.7 ± 51.74	1170.0 ± 50.39*	1176.1 ± 51.04*
HSR (m)	7028.2 ± 823.42	7261.8 ± 905.31*	7186.8 ± 853.76
Sprint (m)	1752.3 ± 381.45	1753.4 ± 367.06	1720.8 ± 332.93
NHIR	598.3 ± 64.06	612.4 ± 68.80*	604.7 ± 62.30

*Significantly different from DC.

*TD* total distance, *TD/min* relative total distance, *HSR* high-speed running distance, *NHIR* number of high-intensity runs.

The data of match performance and physical match activity with reference to 5-match periods are presented in Table 2. Dividing the analysed period into 5-match intervals demonstrated that the average number of collected points by NC in their first 5 games (period IV) was significantly greater (*p* = 0.018, ES = 0.48) than that obtained by DC in their last 5 games (period III). Similarly, TD covered during period IV was significantly longer when compared to periods I (*p* = 0.030, ES = 0.45), II (*p* = 0.004, ES = 0.52) and III (*p* = 0.015, ES = 0.29). TD/min in period IV was significantly greater than that in periods II (*p* = 0.006, ES = 0.52) and III (*p* = 0.028, ES = 0.40). The HSR distance for period IV was significantly longer than that for periods II (*p* = 0.021, ES = 0.44) and III (*p* = 0.049, ES = 0.42). No significant differences in match performance and physical match activity were noted among periods I, II and III (teams led by DC) or between V and VI (after the fifth game of NC).

**Table 2:** Average points collected per game and physical match performance variables (mean ± SD) during 5-match periods for dismissed and new coaches.

	Dismissed coach			New coach		
	I	II	III	IV	V	VI
Average points collected	1.29 ± 1.36	1.09 ± 1.28	0.83 ± 1.14	1.45 ± 1.35^**#**^	1.21 ± 1.28	1.20 ± 1.29
TD (km)	111.9 ± 4.6	111.6 ± 4.3	111.8 ± 4.2	113.7 ± 4.1*^†#^	112.9 ± 4.4	112.3 ± 4.2
TD/min (m/min)	1162.3 ± 50.7	1150.0 ± 53.7	1156.7 ± 50.1	1175.9 ± 45.7^**†#**^	1169.4 ± 51.1	1162.9 ± 54.8
HSR (m)	7056.2 ± 819.2	7004.4 ± 858.6	7040.6 ± 80.0	7399.1 ± 939.0^**†#**^	7284.2 ± 865.8	7052.9 ± 881.6
Sprint (m)	1762.4 ± 386.5	1792.3 ± 401.6	1718.6 ± 357.3	1793.4 ± 405.0	1747.4 ± 359.0	1708.4 ± 320.4
NHIR	600.6 ± 65.6	596.6 ± 65.7	600.2 ± 61.2	621.8 ± 73.0	613.3 ± 64.8	598.7 ± 66.5

I: average points collected by dismissed coaches in a period between match -15 and -10; II: between match -10 and -5; III: in last 5 matches; IV: average points collected by new coaches in their first 5 matches; V: in a period between 5 and 10 match; VI: between match 10 and 15.

*Significantly different from I.

^†^Significantly different from II.

^#^Significantly different from III.

## Discussion

The main purpose of this research was to identify potential changes in match results and physical match performance of soccer teams before and after the coach turnover with reference to the teams led constantly by the same coach. The major finding of the current study is that there is a short-term positive effect on the match outcome and physical match performance after mid-season coach turnover. However, this effect disappears after the first 5 to 10 games of the new coach. Interestingly, the highest value of average collected points was achieved by teams where coaches were unchanged during the analysed period of time. The analysis of the physical match performance revealed that total distance and relative total distance of teams led by NC and UnC were significantly longer in comparison to DC. Furthermore, the values of HSR distance and NHIR significantly increased after hiring the NC.

The phenomenon of a coaching carousel still exists in the top soccer leagues. Tozetto et al.^1^ demonstrated that the survival probability of coaches in the top Brazilian soccer league to hold their job for all seasons was approximately 26%. During the analysed period of time in Polish Ekstraklasa, the ratio of coaches who kept their position until the end of the season varied between 50 and 56% depending on the season. However, at this time, 7 clubs decided to change their coach even twice within one season. Because a new coach needs a certain amount of time to implement his principals, tactical behaviours, style of leadership and type of training, this number of turnovers seems to be exaggerated^29^. The coach’s dismissal decision is usually made after a certain number of consecutive matches during which team performance sharply declines. This period of time was previously defined as approximately two months^30^ or twenty games^30^. In Polish Ekstraklasa, this period seems to be even shorter (Fig. 1) because the results of DC for 10 games prior to dismission remained constant. However, after that time-point, the team’s performance started to decline with the highest dynamics in the last three games. The average number of points collected by DC in game − 1 was only 0.27 ± 0.70 points per match. These findings are consistent with results presented by Zart and Gullich^23^, who reported an outcome decline in the last 8 games of DC with severe performance collapse in the last two rounds before coach turnover.

Simple comparative analysis of the last 15 games of DC prior to dismission and the first 15 games of NC could lead to obvious conclusions that after coach turnover, teams improve their results and physical match performance. However, a more detailed analysis consisting of the division of the 15 games into 5-match periods provides more interesting observations. The average number of points collected by DC in the last 5 matches (period III) prior to dismission was 0.83 points per game, whereas NC collected 1.45 points per match in their first 5 games. The short-term positive influence on the team results after coach turnover is consistent with previous research. Lago-Peñas^21^ reported that in the Spanish soccer league, teams’ performance significantly improves immediately after changing the coach. This shock effect is probably related to increased players’ motivation after the coach’s dismission because the period of a few days seems to be insufficient for new coaches to implement new soccer-specific behaviours of the team^21^. Nevertheless, when the comparison before and after termination is done over 10, 15 or 20 matches, this effect no longer exists^21^. The expiring effect was confirmed in our research, where DC in period I collected more points than NC in periods V and VI. Similar conclusions were reported by Arrondel et al.^31^, who found a slight, short-term improvement in French Ligue 1 teams’ performance after hiring the new coach. In the mid- to long-term (after 10 games), this effect was insignificant. Furthermore, the longitudinal analysis carried out on the top European leagues demonstrated that beneficial effects of the coaching change was absent after 15–20 games^32^. Due to the lack of long-term benefits, mid-season coach dismissions seem to be questionable. This statement was supported by analysis performed on Belgian^30^ and German^22^ leagues, where authors did not observe any significant effect of coach turnover on the teams’ performance. Thus, considering the various areas of club functioning (including sport and financial aspects), leaving the head coach in his position (even despite the crisis) may represent the most effective solution. The highest average number of gathered points by UnC (1.52 points per game) seems to confirm this assumption.

Because the final game result could often be related to physical performance^33^, it seems justified to explore the impact of a new coach on physical match activity. In the available literature, research investigating the impact of coach turnover on physical activity during official matches is limited. Previous studies demonstrated that such contextual variables as game location^34^, match outcome^35^ or opponent level^36^ could affect the physical match performance. A recent study of Augusto et al.^37^ considered the coach turnover as another potential contextual variable. In this research the analysis of a single team indicated that coach replacement negatively affect the match high-intensity activities. Guerrero-Calderon et al.^24^ analysed physical performance during matches and training sessions over an 8-week period (4 weeks before and 4 weeks after coach dismission) in three teams from different levels of competition in Spain. Although the authors found an insignificant trend of higher values of physical variables when playing with DC, the general data analysis showed comparable physical match performance before and after changing the coach. Similarly, Castellano and Casamichana^38^ did not find any significant differences in players’ match locomotion after changing the coach. These results are in contrast with data presented in the current study, where significant improvements in TD, TD/min, HSR distance and NHIR were noted immediately after coach turnover. The increased physical match performance could be affected by the previously mentioned enhanced motivation. Substitute players try to demonstrate their importance for the team, whereas first squad players must prove that they deserve to keep their position^24^.

The new coach effect on the match running performance does not last longer than a few games. Significant improvements in locomotion variables in the current study were observed only in the first 5 games of NC (period IV). The physical performance in subsequent matches did not differ significantly from DC. It is worth emphasizing that registered values of NHIR and sprint distance between matches 10 and 15 of NC (period VI) were even lower when compared with the last matches of DC (period III). Previous studies have indicated the importance of these parameters as highly predictive for the game result in professional soccer^39^. Similar to the number of collected points, the gradual decline in physical match performance may have been caused by the stabilization of the level of motivation. When the new coach’s psychological effect disappears, the manager’s ability to lead the team is a crucial variable of team performance^31^. Thus, the fact that the decline in locomotion parameters is out of proportion with the number of collected points by NC could serve as an inspiration for further studies. Therefore, future research considering the topic of coach turnover in soccer should involve tactical, technical and physical performance and descriptive variables regarding coaches’ experience, age or previous successes. In addition, squad rotations made by new coaches with special reference to the number of previously reserve players moved to the first squad should be further investigated.

This study investigated the influence of mid-season coach turnover on match outcome and physical match performance with reference to teams that were led continuously by the same coaches. Despite several important findings, some limitations are worth mentioning. In the presented analysis, contextual variables, such as game location, quality of opponent or game status, were not taken into consideration. Although, the *p* values indicated several significant differences, the ES values were rather small. Therefore, the results of this study should be interpreted with caution.

## Conclusions

After a few games ended with unsatisfactory results, the club authorities often decide to terminate the contract of the first coach. The current study results showed that changing the coach during the soccer season may result in short-term improvement in team results and physical match performance. However, after a period of approximately 5 games, this effect disappears. Therefore, changing the coach to improve team results may not always be an appropriate solution. The highest number of collected points per game are obtained by coaches who lead their teams for several seasons. The results mentioned above suggest that the selection and hiring of an appropriate coach suitable for the specific team and providing sufficient time for longitudinal work could positively affect match performance in professional soccer.

## Data Availability

The data used for this study was acquired from a third-party, https://tracabportal.azurewebsites.net/login. The data was provided under scientific cooperation with a football clubs currently appearing in Ekstraklasa. The authors’ ethical approval also prevents them from sharing any data in any way that could be re-identified. The metadata would allow someone else to re-identify teams and possibly players. However, access to the data should be possible from the third-party. The data acquired were so called ‘excel dumps’ of player statistics per match. Access to the data can be organised by contacting Match Analysis info@chyronhego.com.
